# Neuroprotection by Exendin-4 Is GLP-1 Receptor Specific but DA D_3_ Receptor Dependent, Causing Altered BrdU Incorporation in Subventricular Zone and Substantia Nigra

**DOI:** 10.1155/2013/407152

**Published:** 2013-11-21

**Authors:** A. Harkavyi, N. Rampersaud, P. S. Whitton

**Affiliations:** Department of Pharmacology, The School of Pharmacy, University of London, 29-39 Brunswick Square, London WC1N 1AX, UK

## Abstract

Glucagon-like peptide-1 receptor (GLP-1R) activation by exendin-4 (EX-4) is effective in preclinical models of Parkinson's disease (PD) and appears to promote neurogenesis even in severely lesioned rats. In the present study, we determined the effects of EX-4 on cellular BrdU incorporation in the rat subventricular zone (SVZ) and substantia nigra (SN). We also determined the specificity of this effect with the GLP-1R antagonist EX-(9-39) as well as the potential role of dopamine (DA) D_3_ receptors. Rats were administered 6-OHDA and 1 week later given EX-4 alone, with EX-(9-39) or nafadotride (D_3_ antagonist) and BrdU. Seven days later, rats were challenged with apomorphine to evaluate circling. Extracellular DA was measured using striatal microdialysis and subsequently tissue DA measured. Tyrosine hydroxylase and BrdU were verified using immunohistochemistry. Apomorphine circling was reversed by EX-4 in lesioned rats, an effect reduced by EX-4, while both EX-(9-39) and NAF attenuated this. 6-OHDA decreased extracellular and tissue DA, both reversed by EX-4 but again attenuated by EX-(9-39) or NAF. Analysis of BrdU+ cells in the SVZ revealed increases in 6-OHDA-treated rats which were reversed by EX-4 and antagonised by either EX-(9-39) or NAF, while in the SN the opposite profile was seen.

## 1. Introduction

Evidence suggests that the glucagon-like peptide-1 receptor (GLP-1R) agonist exendin-4 (EX-4), used in the treatment of type 2 diabetes mellitus, also displays neuroprotective properties in multiple cellular and *in vivo* models of neurodegenerative disorders. Evidence of its potential for the treatment of these neuropathologies has been accumulated rapidly [1–5]. The drug appears to be well tolerated and its use in the clinic, albeit for a different indication, obviates many of the obstacles seen with other putative treatments for PD. A key factor with EX-4 is that despite being a relatively large peptide it readily enters the CNS [6].

EX-4 is a potent agonist at mammalian GLP-1Rs and promotes insulin secretion from beta islet cells. Additionally, EX-4 protects beta cells from cytotoxic insults [7] and also promotes their proliferation and neogenesis from precursors [8, 9]. This suggests that similar mechanisms could be responsible for neuronal cell survival in animal models of neurodegenerative disorders in which EX-4 has been shown to be effective. Neuroprotective effects have been shown *in vitro* to be GLP-1R dependent by the use of the GLP-1R selective antagonist EX-(9-39) and also more recently in GLP-1R knockout mice [5]. A possible mechanism of action for EX-4 is that it causes a reduction of deleterious inflammation [4]. EX-4 blocks MPTP-induced microglial activation and reduced expression of matrix metalloproteinase-3 [4]. However, EX-4 has also been shown to stimulate neurogenesis in the adult rat, which constitutes a possible means by which the peptide might restore function in a damaged system [1]. 

In the context of PD, a potential influence could also be activation of DA D_3_ receptors, which are thought to be involved in stimulation of neurogenesis, as demonstrated in the rat 6-OHDA model of PD [10, 11]. We have, therefore, decided to determine whether the neuroprotective effect of EX-4 is selectively GLP-1R mediated by use of EX-(9-39) in our 6-OHDA model. Secondly, since stimulation of DA D_3_ receptors has been shown to protect DA neurons against 6-OHDA-induced damage and promote neuronal survival as well as stimulate neurogenesis in the subventricular zone (SVZ) [10, 11], we wanted to evaluate the possible role of D_3_ receptors in EX-4-mediated neuroprotection by using nafadotride (NAF), a selective D_3_ receptor antagonist. Finally, we wished to determine the effects of EX-4 on BrdU+ cell numbers in the SVZ and SN of 6-OHDA-lesioned rats and how this might be altered by EX-(9-39) or NAF.

## 2. Experimental Procedures 

All drugs and reagents were purchased from Sigma-Aldrich Ltd., UK, unless otherwise specified.

### 2.1. Animals

Male Wistar rats (250–300 g) were purchased from Harlan, UK, and housed under standard conditions in an animal care facility with controlled temperature, humidity, and fixed light/dark cycles. Animals were group housed and had free access to food and water. All experimental procedures were carried out in accordance with institutional and home office regulations (1986 Scientific Procedures Act, UK) and the Helsinki Agreement.

### 2.2. Stereotaxic Surgery

Rats were anaesthetised using 5% isoflurane (Abbott, UK) v/v in O_2_ for induction (1.5% for maintenance) and then secured on a stereotaxic frame (David Kopf, USA). Injection was made using a 10 *μ*L Hamilton syringe. Prior to surgery, animals were injected with pargyline (50 mg/kg) and desipramine (25 mg/kg) intraperitoneally (i.p.). This was done to minimize the metabolism of 6-OHDA and to preserve the noradrenergic system, respectively. The animals were, then, injected with 6-OHDA hydrochloride (8 *μ*g/2 *μ*L) dissolved in saline containing 0.2% ascorbic acid into the left medial forebrain bundle (from bregma in mm; A −4.3, L 1.4, and V 8.2). Infusion was performed at a rate of 1 *μ*L per minute, and a 5-minute period was allowed for diffusion of the toxin after which the syringe was slowly retracted.

### 2.3. Drug Administration

Animals were given EX-4 i.p. (0.5 *μ*g/kg) or saline twice a day (10 am and 5 pm), for a period of one week, seven days after the administration of 6-OHDA. Rats cotreated with EX-9-39 received i.p. injections of the compound 1 *μ*g/kg or saline twice a day 5 min prior to EX-4 injection. In another group of rats NAF was given i.p. (1 mg/kg), a dose that is very largely D_3_ selective, or saline twice daily, also 5 min prior to EX-4. The dose of NAF was based upon studies also using this dose with apparent D_3_ selectivity [12, 13]. In a separate set of treatment groups BrdU was injected to investigate if lesions and treatment with EX-4 altered cell proliferation in the SVZ. BrdU is a brominated thymidine analog able to incorporate into the DNA of actively dividing cells and is currently a frequently used measure of presumed neurogenesis. Different treatment groups were compared to check how EX-4 affects cell proliferation. Animals treated with BrdU received i.p. injections of the compound (50 mg/kg twice a day) 10 min after injection of EX-4 and the antagonists. These groups were used exclusively for BrdU immunohistochemistry and at the end of the BrdU treatment were allowed a 4-day “washout” period to excrete unincorporated BrdU.

### 2.4. Apomorphine Circling

Fourteen days after surgery, animals were injected with 0.05 mg/kg of apomorphine subcutaneously (s.c.) in saline, and after 15 minutes each rat was placed in a circular test arena. Two minutes later, the number of turns was counted per 120-second period. The number of turns is indicative of the presence of the neurotoxic lesion. We have used longer assessment periods in the past but found that these simply revealed quantitatively larger but qualitatively very similar numbers of turns between groups and were, therefore, deemed an unnecessary use of time. This accords with procedures we have tested and used previously [2, 3].

### 2.5. *In Vivo* Microdialysis

Surgery was performed in these rats 14 days after 6-OHDA injection. Animals were anaesthetised with isoflurane (5% induction v/v in O_2_ and 1.5% maintenance) and then secured in a stereotaxic frame. Microdialysis probes, constructed as described previously and with a membrane length of 4 mm (Whitton et al., 1991), were bilaterally implanted into the striatum (mm from bregma A +0.2, L 3, and V 8); dental screws were placed within but not penetrating the skull; the assembly was fixed solidly with dental acrylic (DuraLay, Reliance Dental MfG. Co.). After surgery, animals were placed in individual microdialysis cages and allowed to recover for 24 hours before dialysis. The following day rats were perfused with artificial cerebrospinal fluid (aCSF) (2.5 mM KCl, 125 mM NaCl, 1.18 mM MgCl_2_·6H_2_O, and 1.26 mM CaCl_2_) pH 7.4 at a rate of 1 *μ*L/min using Harvard Apparatus model 22 syringe infusion pumps. After a stable baseline was established, around 1 hour, samples were collected every 30 minutes and after sample four “normal” aCSF was switched to one containing 100 mM KCl, with Na^+^ concentration reduction to maintain osmolarity, for 30 minutes before returning to normal aCSF for the rest of the experiment. Samples were frozen immediately at −80°C and analysed for DA within one week using high-performance liquid chromatography (HPLC) with electrochemical detection [14]. 

### 2.6. DA Determination

The HPLC system comprised a P580 Dionex, piston pump (mobile phase: sodium acetate; 90 mM, citric acid; 35 mM, EDTA, 0.34 mM; 1-octane-sulfonic acid, 0.06 mM and 5.5% methanol with pH was adjusted to 4.2 using citric acid, flow rate of 0.65 mL/min) connected to a Triathlon refrigerated (4°C) autosampler (Spark-Holland, The Netherlands) and a C18 reverse phase column maintained at 35°C (ODS 3 WM, 4.6 mm I.D. × 100 mm; Rainin Dynamax Instruments Co. INC., USA) and protected by a Microsorb guard column (C18 5 WM, 4.6 mm I.D. × 15 mm, Rainin Dynamax Instruments Co. INC., USA). Detection was made with an Antec-Intro electrochemical detector (Antec Leyden BV, The Netherlands) fitted with a VTO3 flow cell (Vcell +625 mV filtered to 5 abu with range set on 0.5 nA/volt). Data capture was achieved and analysed by a PC system (Dell Corporation, USA) equipped with Chromperfect for Windows software (Justice Innovations Chromatography Data Systems, CA, USA). DA peak areas were converted to amounts using the external standard method. 

### 2.7. Termination and Tissue Processing

Animals were always sacrificed on completion of microdialysis (two weeks after 6-OHDA surgery). Rats were lightly anaesthetised with 5% isoflurane v/v in O_2_ in order to minimise stress and decapitated, and the brains were rapidly removed, flash frozen on dry ice, and stored at −80°C until use. One day before histology, frozen brains were taken from −80°C storage and stored at −20°C overnight to facilitate dissection and cryostat sectioning. Striata were removed using a stainless steel punch, placed into microcentrifuge tubes containing 1 mL ice cold 0.01 M PBS and homogenised for DA determination. Homogenates were centrifuged at 9000 ×g for 15 minutes, and duplicates of 50 *μ*L aliquots of the supernatant were treated with 0.2 M perchloric acid containing ascorbic acid (0.2 *μ*M) and EDTA (0.2 *μ*M) to precipitate the cell debris. The 50 *μ*L homogenate and 10 *μ*L perchloric acid mixtures were centrifuged again at 9000 ×g for 15 minutes at 4°C, and 25 *μ*L of this supernatant was injected in the HPLC system to determine tissue DA concentration in pg/gram of striatal tissue.

### 2.8. Histology

Slide mounted 12 *μ*m thick cryostat sections from frozen brain blocks were removed from the freezer and allowed to equilibrate to room temperature for 30 minutes before fixation in 4% w/v paraformaldehyde containing 1% w/v glutaraldehyde in deionised water for 5 minutes at 0°C. Following rinsing in 0.01 M PBS for 5 minutes, sections were dehydrated through graded ethyl alcohol concentrations, and endogenous peroxidase activity was blocked by incubation in 0.3% H_2_O_2_ in methanol for 10 minutes. The sections were then rehydrated, and nonspecific immunoreactivity was blocked using 10% swine serum in 0.01 M. PBS for 10 minutes. Sections were then incubated in primary antibody (anti-TH IgG raised in rabbit at 1 : 1000 in 0.01 M PBS) for 15 hours at 4°C. After rinsing, the sections were incubated sequentially in biotinylated swine anti-rabbit antibody 1 : 500 in 0.01 M. PBS and ABC (Vector Labs Ltd.) complex was applied for 30 minutes at room temperature following the manufacturer's instructions. Immunoreactivity was visualized through incubation in 0.5 mg/mL 3′,3-diaminobenzidine (DAB), containing 0.009% H_2_O_2_ in 20 mL of DAB solution, for 2 minutes at room temperature. The sections were counterstained in Harris haematoxylin, dehydrated, cleared, and mounted for examination. Sections were viewed under a light microscope using 5x magnification. Digital images were captured using a Leica DC500 system and the manufacturer's software.

For BrdU histology, slide mounted 12 *μ*m thick cryostat sections from frozen brain blocks were removed from the freezer and allowed to dry for 24 hours before fixation in 75% acetone and 25% absolute alcohol for 5 minutes. Slides intended for BrdU immunodetection were also preincubated in 2 M HCl for 30 min at 37°C to facilitate antigen retrieval. The same procedure was followed as for TH immunodetection. The BrdU antibody used was a goat monoclonal raised against BrdU (1/1000) followed by a secondary rabbit anti goat-polyclonal antibody with a biotin tag (1/500). BrdU staining was quantified, by an investigator unaware of the treatment protocol, by manually counting cells over a set distance along the lateral ventricular wall viewing sagittal sections. 

### 2.9. Statistics

Data obtained from apomorphine tests, tissue DA, TH, and BrdU immunohistochemistry were subjected to one-way analysis of variance (ANOVA) and post hoc Bonferroni multiple comparison tests to compare difference between selected treatments. Microdialysis data were subjected to a two-way ANOVA with a post hoc Bonferroni multiple comparison test. In both cases, data were expressed as mean ± standard error of the mean. GraphPad Prism 5 software was used for all of the statistical manipulations. 

## 3. Results

### 3.1. Apomorphine Circling

**Figure 1:**
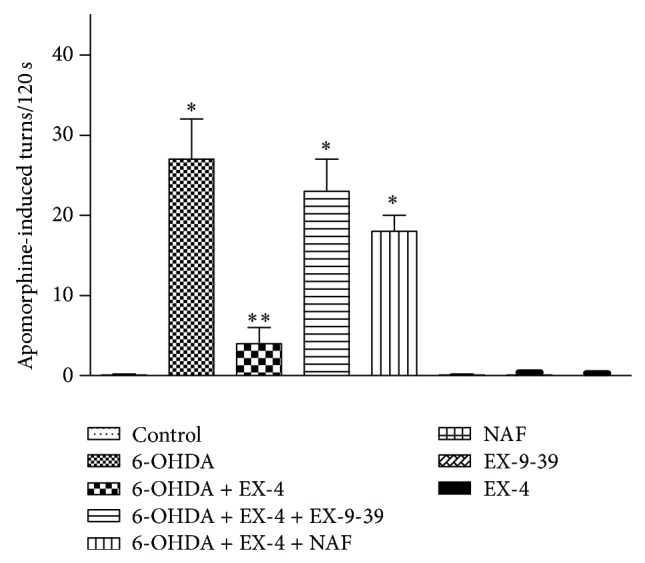
Effect of EX-4 on apomorphine-induced circling in the presence of 1 *μ*g kg^−1^ EX-9-39 and 1 mg kg^−1^ NAF. Both antagonists were coadministered with EX-4 twice daily for seven days, one week after toxin injection. Circling was counted for 120 s 15 min after 0.5 mg kg^−1^ apomorphine injection. Results were analyzed using one-way ANOVA (*F* = 34.27; *P* < 0.0001) and a post hoc Bonferroni test to compare differences between groups. ∗*P* < 0.001 compared to shams, EX-4, NAF, and 6-OHDA + EX-4 groups; ∗∗*P* < 0.01 compared to shams, 6-OHDA, EX-4, EX-(9-39), NAF, 6-OHDA + EX-4 + EX-(9-39), and 6-OHDA + EX-4 + EX-(9-39).

Apomorphine-induced circling was tested in treated rats (Figure 1). The 6-OHDA-lesioned rats treated with apomorphine two weeks after surgery made intensive turning behavior compared to sham animals that do not show turns (Figure 1). Circling was significantly reduced by cotreatment with EX-4, an effect attenuated by both EX-9-39 and NAF (Figure 1). Neither of the antagonists or EX-4 administered alone to naïve animals had any effect on apomorphine circling.

### 3.2. Tissue and Extracellular DA Levels

**Figure 2:**
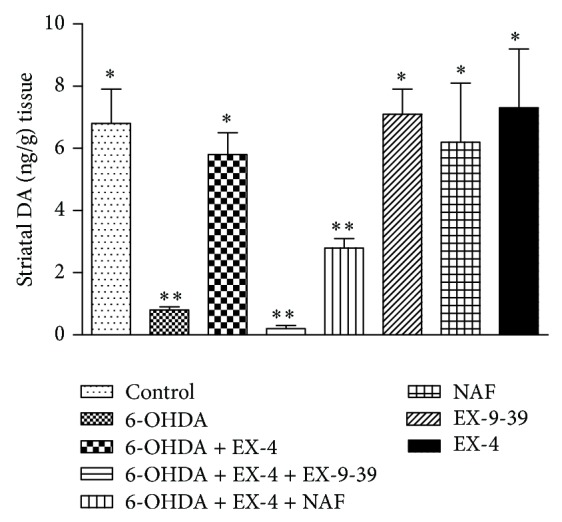
Effect of 0.5 *μ*g kg^−1^ EX-4 alone or in the presence of EX-9-39 or NAF on striatal tissue DA. Both antagonists were coadministered with EX-4 twice daily for seven days, one week after toxin injection. Results were analyzed using one-way ANOVA (*F* = 17.13; *P* < 0.001) and a post hoc Bonferroni test to compare differences between groups. Results were analyzed using one-way ANOVA (*F* = 34.27; *P* < 0.0001) and a post hoc Bonferroni test to compare differences between groups. ∗*P* < 0.001 compared to 6-OHDA, 6-OHDA + EX-4 + EX-(3-39), and 6-OHDA + EX-4 + NAF groups; ∗∗*P* < 0.01 compared to shams, 6-OHDA, 6-OHDA + EX-4, EX-4, EX-(9-39), and NAF.

In sham groups the mean DA content was 6.8 ± 1.1 pg/g of tissue while in 6-OHDA-only groups there was a substantial reduction in DA levels (0.77 ± 0.2 pg/g). In groups cotreated with EX-4 tissue DA was restored to 5.2 ± 0.33 pg/g, an effect reversed by both EX-9-39 (0.19 ± 0.1 pg/g) and, to a lesser extent at the doses employed here, by NAF (2.6 ± 0.53 pg/g); both results were significantly different from 6-OHDA + EX-4 (Figure 2). Animals treated with either EX-4, EX-9-39, or NAF alone did not show significant changes in their striatal DA tissue content compared to control groups.

**Figure 3:**
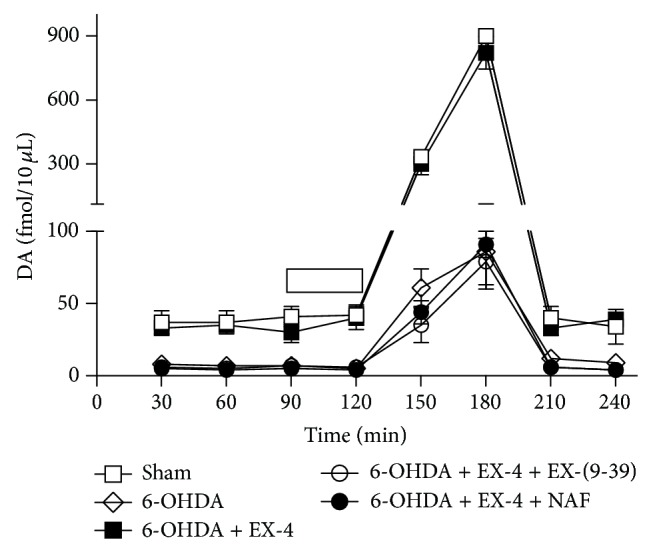
Effect of EX-(9-39) or NAF on recovery of striatal extracellular DA by EX-4 in 6-OHDA-lesioned rats. Both antagonists were coadministered with EX-4 twice daily for seven days, one week after toxin. Bar at 90–120 min indicates infusion of 100 mM K^+^. Results were analyzed using two-way ANOVA (*F* = 13.32 between treatments and 43.52 overtime) and a post hoc Bonferroni multiple comparisons test. All basal and evoked 6-OHDA and EX-4 + either EX-(9-39) or NAF groups were significantly different (*P* < 0.01) from shams or 6-OHDA + EX-4 at all time points.

Basal and K^+^-evoked striatal extracellular DA were assessed using *in vivo* microdialysis, with DA release shown as fmol/10 *μ*L of sample. Basal DA levels in control groups were 39 ± 1.2 fmol/10 *μ*L, increasing to 1108 ± 113 fmol/10 *μ*L when stimulated with 100 mM KCl and rapidly returning to baseline on removal of this stimulus (Figure 3). In 6-OHDA-only-treated rats basal DA was reduced to 8 ± 3 and K^+^-induced DA release decreased to 188 ± 34 fmol/10 *μ*L. It is noteworthy that the decrease in extracellular DA was proportionately less than tissue DA in lesioned animals. Rats given 6-OHDA and then cotreated with EX-4 displayed a significant restoration of both basal (28 ± 9 fmol/10 *μ*L) and K^+^-evoked (780 ± 87 fmol/10 *μ*L) extracellular DA (Figure 3). 6-OHDA rats cotreated with EX-9-39 + EX-4 displayed drastically reduced levels of both basal and K^+^-induced extracellular DA compared with 6-OHDA and EX-4 groups (Figure 3), as did EX-4 rats cotreated with NAF (basal 5 ± 1 fmol/10 *μ*L and K^+^-induced 125 ± 21 fmol/10 *μ*L; Figure 3). DA levels in sham animals treated with EX-4, EX-9-39, or NAF only were statistically not different from the sham groups (data not shown).

### 3.3. TH Content

**Figure 4:**
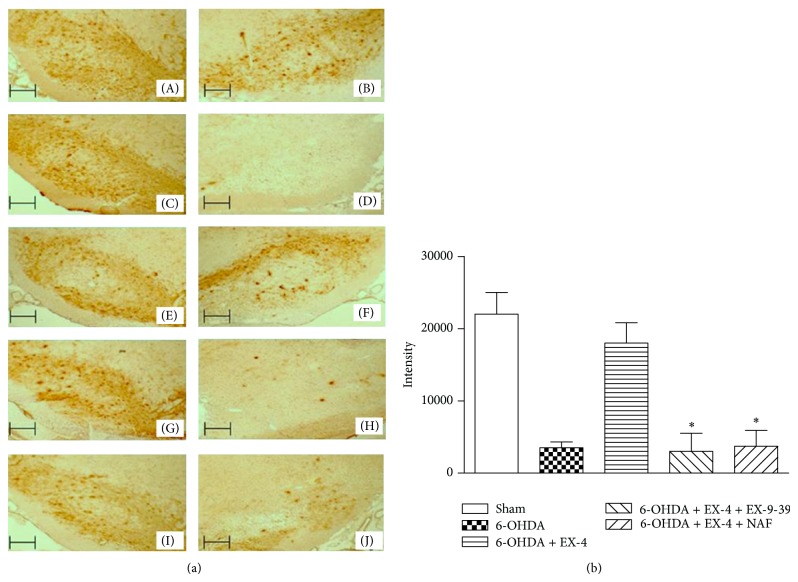
(a) Representative photomicrographs of 12 *μ*m cryostat sections through the rat SN immunoassayed for TH. Nigral TH cell bodies appear dark brown in colour visualised using DAB staining; (A) and (B) represent right and left untreated SN, respectively; (D) represents 6-OHDA lesion assessed after two weeks compared to untreated side (C); (F) shows the effect of 0.5 *μ*g/kg exendin-4 in a 6-OHDA-treated animal one week after lesion compared to untreated SN (E); (H) and (J) represent the effect of EX-9-39 or NAF coadministration, respectively, in 6-OHDA- and EX-4-treated animals compared to corresponding untreated sides (G) and (I). Scale bars—100 *μ*m. (b) represents quantified data from immunohistochemical analysis of SN for TH. DAB staining was quantified using Lucia G image analysis software based on colour intensity. Results analyzed using one-way ANOVA and a post hoc Bonferroni test to compare differences between groups. ∗indicates significant differences compared to 6-OHDA + EX-4 group (ANOVA *F*[4, 25] = 16.99, ∗*P* < 0.05, *n* = 6 per group, confidence interval set at 95%).

TH staining is shown both qualitatively (photomicrographs) and quantitatively (Figures 4(a) and 4(b)). SN region was visualized using immunohistochemistry with DAB staining as a quantifiable measure. Control groups had a high level of TH expression (colour intensities of 21532 ± 2853) while groups treated with 6-OHDA and tested after two weeks showed an approximate 80% decrease in TH content compared to controls (Figure 4), an effect reversed by EX-4 (18293 ± 2620 units). In 6-OHDA and EX-4 groups which also received EX-9-39, TH decreased to 3225 ± 1893 units, while after cotreatment with NAF intensities were decreased to 5680 ± 2854 units (Figure 4). Groups which were treated with EX-4, EX-9-39, or NAF only were not significantly different from controls (data not shown). 

### 3.4. BrdU Incorporation

**Figure 5:**
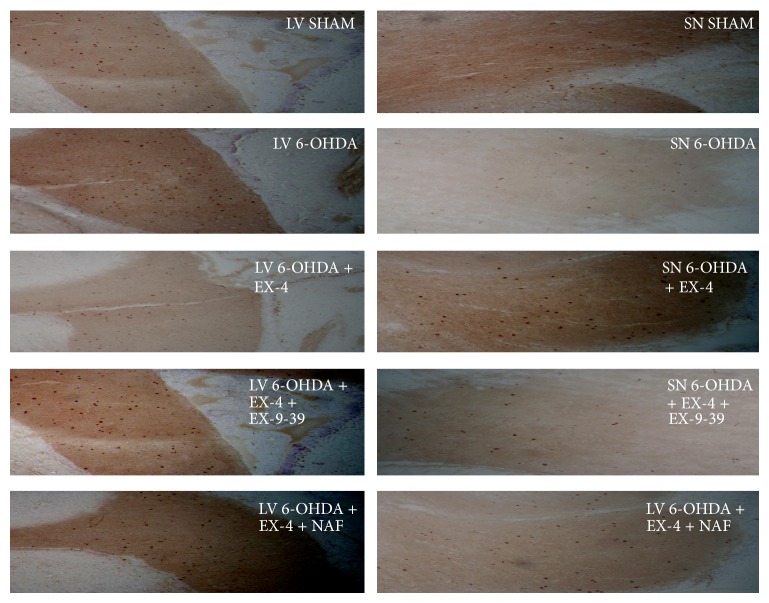
Representative examples of selected 12 *μ*m cryostat sections through the rat SVZ and SN immunoassayed for BrdU. BrdU+ cells appear dark brown visualised using DAB staining. SVZ sections were taken from the lateral ventricle (LV).

**Figure 6:**
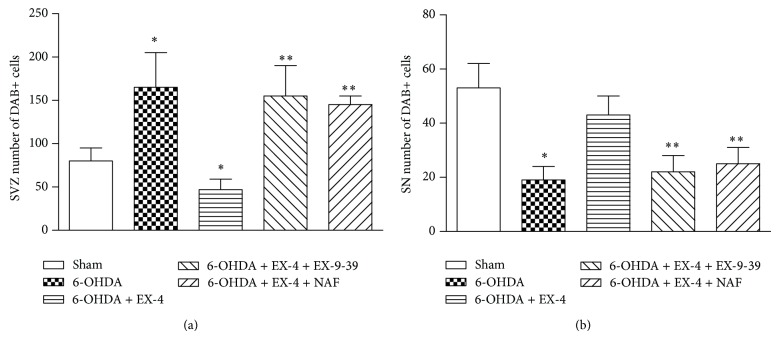
Quantified histograms of DAB-stained cell counts from 6 sections from each of the brains in the treatment group (*n* = 6) were used for analysis. DAB cell counts along the wall of the SVZ (a) or SN (b) were carried out manually. Results were analyzed using one-way ANOVA (SVZ *F* = 5.83; SN *F* = 3.847) and a post hoc Bonferroni test to compare relevant treatment groups. ∗*P* < 0.01 indicates significant difference compared to sham and 6-OHDA + EX-4. ∗∗*P* < 0.01 indicates significant difference compared to 6-OHDA and EX-4 treatment.

BrdU was coadministered to treatment groups to investigate cell proliferation in the rat SVZ and SN while DAB staining was used to visualise BrdU antibody binding. Results were expressed as numerical density calculated as numbers of DAB positive cells per section. In the SVZ, 6-OHDA-only-treated animals showed a significant increase in BrdU+ cells, which was reversed by EX-4. In turn, the effects of EX-4 were markedly attenuated by EX-(9-39) (Figures 5 and 6). In the SN, the reverse was seen. 6-0HDA dramatically reduced the count of BrdU+ cells, and this was largely reversed by EX-4. Both antagonists decreased the effect of EX-4, resulting in reductions in BrdU+ cells similar to that seen with 6-OHDA alone (Figures 5 and 6). 

## 4. Discussion

Many studies, both *in vitro *and now *in vivo*, strongly indicate the therapeutic potential of EX-4 in neurodegenerative disorders where GLP-1Rs appear to be the key in mediating these effects. The present report indicates that GLP-1Rs as well as DA D_3_ receptors are required for neuroprotective effect of EX-4 in the 6-OHDA rat model of PD. Thus, when the specific antagonist of GLP-1R EX-(9-39) was coadministered with EX-4 in 6-OHDA rats, animals displayed increased contralateral circling, reduced extracellular as well as tissue DA levels, and also decreased SNc TH expression, a complete reversal of the previously observed effects of EX-4 [2].

GLP-1Rs are also expressed throughout the central nervous system, with binding sites identified in both the human and the rat brain [16], and are produced by neuronal cells in discrete regions of the CNS [17]. More recently, the neuroprotective role of GLP-1Rs was demonstrated in an MPTP mouse model of PD [7]. EX-4 was able to protect normal mice against MPTP-induced damage of the SN, whereas no effect was observed in GLP-1R deficient mice, indicating that the GLP-1R is instrumental for EX-4-mediated neuroprotection. The manner in which EX-4 or GLP-1 itself is protective is yet unclear. However, broadly, it seems that there may be a rescue of dying neurons that are not yet “fatally” damaged [2], quite possibly as a result of the well-known anti-inflammatory properties of these molecules. Alternatively, it has been suggested that GLP-1 and EX-4 are able to promote *de novo* neurogenesis as indicated by the findings of Bertilsson et al. [1].

In our studies we have observed increased accumulation of BrdU+ cells in the SVZ of 6-OHDA-treated animals, an effect which was reduced significantly when animals were given EX-4, while in the SN the opposite effect was seen with reduced BrdU+ cells in 6-OHDA-treated rats and a reversal with EX-4. The effects of EX-4 were themselves completely reversed by EX-(9-39), indicating a GLP-1 receptor specific primary action. 

Additionally, the effects of EX-4 in the SVZ and SN were attenuated by NAF, indicating that DA D_3_ receptors play a role in the overall process. D_3_ receptors are localized on DA neurons of the adult SN [10, 11] as well as upon the surface of progenitor cells in the SVZ, where their stimulation appears to elicit maturation and migration of these cells [10, 11, 18]. These observations underpinned our interest in the possible role of these receptors as facilitators of GLP-1R-mediated neuroprotection. Interestingly, evidence has been accumulated from animal models suggesting that D_3_ receptor stimulation is neuroprotective [19]. Selective agonists of this receptor subtype are effective in both MPTP-treated mice [19, 20] and 6-OHDA rat models of PD [10, 11, 21]. Neuroprotective effects of these agents are abrogated in the presence of the D_3_ antagonist A-437203 and also in mice lacking D_3_ receptors [22]. Evidence from both *in vitro* and *in vivo* studies suggests that protective effect of pramipexole, which is a preferential D_3_ agonist used in the treatment of early PD, is dependent on brain-derived neurotrophic factor (BDNF) signaling [19, 23, 24]. *In vivo* evidence demonstrates that infusion of a D_3_ preferring agonist into lateral ventricles doubled cell proliferation in the SVZ and the olfactory bulb whereas no such effect was observed with a D_1_ preferring agonist or in the presence of a D_3_ antagonist [10, 11]. In addition, proliferation of cultured SVZ cells treated with various DA agonists correlated with affinity of the agonist for the D_3_ receptor and was reduced in the presence of an antagonist of this receptor subtype [25]. D_3_ receptor stimulation not only results in SVZ cell proliferation but stimulated cells also display neuron specific markers in both SVZ and the rostral migratory stream [10, 11]. Moreover, a 2-week infusion of a D_3_ preferring agonist into the third ventricle resulted in a sixfold increase in BrdU-labeled cells in the SN which were also TH positive and displayed a neuronal phenotype [11]. This treatment also promoted a behavioural recovery in the 6-OHDA rat model [11]. The linkage between GLP-1R and D_3_ receptors has not been studied directly as far as we are aware but is of obvious interest and has been tentatively suggested [15]. Our choice of the NAF dose was based upon previous studies [12, 13] where there was a clear selectivity for the use of a 1.0 mg/kg. Indeed, in one study a question raised was whether this dose is sufficient to determine the effect of D_3_ receptor blockade [13].

The effects of 6-OHDA on BrdU incorporation into cells in the SVZ are themselves somewhat controversial [26, 27]. Progenitor cell numbers in the SVZ have been observed to decrease in response to dopaminergic denervation [28, 29] while in other studies increases are observed [30, 31]. Evidence for neurogenesis in the SN is also controversial with some studies reporting increased levels of this phenomenon in MPTP models [26] while others using 6-OHDA-treated rats report no evidence of SN neurogenesis [32]. Results obtained from human PD patients demonstrate reduced cell proliferation in the SVZ, and changes in adult neurogenesis resulting from neurodegenerative disorders likely depend on selected neuronal populations affected; and the role of neurogenesis needs further clarification [26, 27]. The effects we observe here could be explained by alterations in the migration of BrdU+ cells between the SVZ and SN. These structures are linked by a dopaminergic pathway [33], stimulation of which would presumably increase DA tone on progenitor cells and augment their proliferation and/or migration. Presumably the converse may occur if the pathway is damaged, such as by DAergic denervation, leading to a potential accumulation of progenitor cells in the SVZ and a decrease in the SN. This is exactly what we have observed and is reversed by EX-4, which is efficacious in preclinical PD models. Whether this contributes to the positive effect of EX-4 is unclear but at the very least, in theory, could play some role in the neuroprotective actions of EX-4 in these models. 

## 5. Conclusions

EX-4 has proven efficacy in preclinical models of PD. More recently evidence suggests that EX-4 has actual therapeutic efficacy, as indicated in a small scale clinical trial [34]. These observations have naturally led to understanding the mechanism of action of EX-4 being of intense interest. The present data suggest that EX-4 reverses the decrease in BrdU+ cells in the SN of lesioned rats. This effect was mirrored by the opposite observations in the SVZ. Since this is blocked by DA D_3_ receptor stimulation one possibility may be that EX-4 alters the migration and/or maturation of progenitor cells. Moreover, our findings with EX-9-39 unambiguously show that these effects are primarily mediated by the GLP-1R.

## Abbreviations

CNS: Central nervous system
DAB: Diaminobenzidine
DA: Dopamine
EX-4: Exendin-4
GLP-1: Glucagon-like peptide-1
GLP-1R: Glucagon-like peptide-1 receptor
HPLC: High-pressure liquid chromatography
6-OHDA: 6-Hydroxydopamine
LV: Lateral ventricle
NAF: Nafadotride
SN: Substantia nigra
SVZ: Subventricular zone
TH: Tyrosine hydroxylase.
